# Hybrid Plasmonic Microring Nano-Ruler

**DOI:** 10.1038/s41598-018-27499-7

**Published:** 2018-06-15

**Authors:** Jing Du, Jian Wang

**Affiliations:** Wuhan National Laboratory for Optoelectronics, School of Optical and Electronic Information, Huazhong University of Science and Technology, Wuhan, 430074 Hubei China

## Abstract

Surface plasmonic polariton (SPP) has attracted increasing interest for its ability of confining light in the subwavelength scale and breaking the diffraction limit. Recently, there have appeared several important developments of SPP applied in plasmon rulers, waveguides and resonators. By combing these concepts we present a novel hybrid plasmonic microring nano-ruler relying on the sensitive hybrid mode property and the microring resonator structure. The designed nano-ruler can measure distance in nanoscale resolution and offer adjustable sensitivity, which exceeds 14.8 as the distance is less than 5 nm by recording the transmission spectra and outstrips 200 dB/nm by observing the shift of output intensity. These demonstrations suggest that hybrid plasmonic microring nano-ruler could be a promising candidate enabling high-resoluation measurement.

## Introduction

Surface plasmonic polariton (SPP) has been widely studied for its ability of confining light in the subwavelength scale, providing the potential to break the diffraction limit^1–5^. The ultra-small light localization can benefit lots of applications and various types of SPP-based devices have attracted increasing interest, such as waveguides, microring resonator filters, lasers, and sensors^5–18^. Several interesting and important developments of SPP were addressed in these prior art works: 1) using coupled plasmon resonances, a new concept of plasmon rulers was proposed to determine nanoscale distances which can be applied in monitoring DNA hybridization and biological processes in living cells by giving a complete picture of time-dependent nanoscale activities and rearrangements^7,8,10^; 2) using combined contributions from SPP and discontinuity of electric field at the interface between two dielectrics with high-contrast refractive index, hybrid plasmonic waveguides achieving subwavelength mode confinement while maintaining the hundred-micrometer propagation distance were reported^5,11–14^; 3) combing hybrid metal-oxide-semiconductor structure^15–17^ with microring resonator, ultracompact hybrid plasmonic microring resonators were presented providing new approaches to develop fully CMOS-compatible plasmonic integrated devices for signal processing and sensing^18^. The attractive idea, i.e. plasmon ruler, can be regarded as a displacement sensor, which gives distance in nanoscale resolution by measuring the spectral shift. Beyond existing schemes of plasmon ruler, one would expect to add diversity of ruler by incorporating new mechanism and structure already employed in other devices like hybrid plasmonic waveguides and ring resonators. For example, the hybrid mode which is sensitive to the width of low-index gap region and the microring structure whose resonance peaks is dependent on the effective mode index could be considered. In this scenario, it might be interesting to combine these features together in a nano-ruler.

In this paper, by combing the concept of plasmon ruler with hybrid mode and microring resonator structure, we design a novel hybrid plasmonic microring nano-ruler to measure the distance in nanoscale between the microring and metal layer. Two working principles of the proposed nano-ruler are simulated and discussed. In the first working principle, sensitivity above 14.8 for distance below 5 nm by recording the transmission spectra is achieved. In the second working principle, sensitivity above 200 dB/nm for distance below 5 nm by observing the output intensity is captured. In addition, measuring range of the designed nano-ruler is also discussed.

## Mode In Hybrid Plasmonic Waveguide

**Figure 1:**
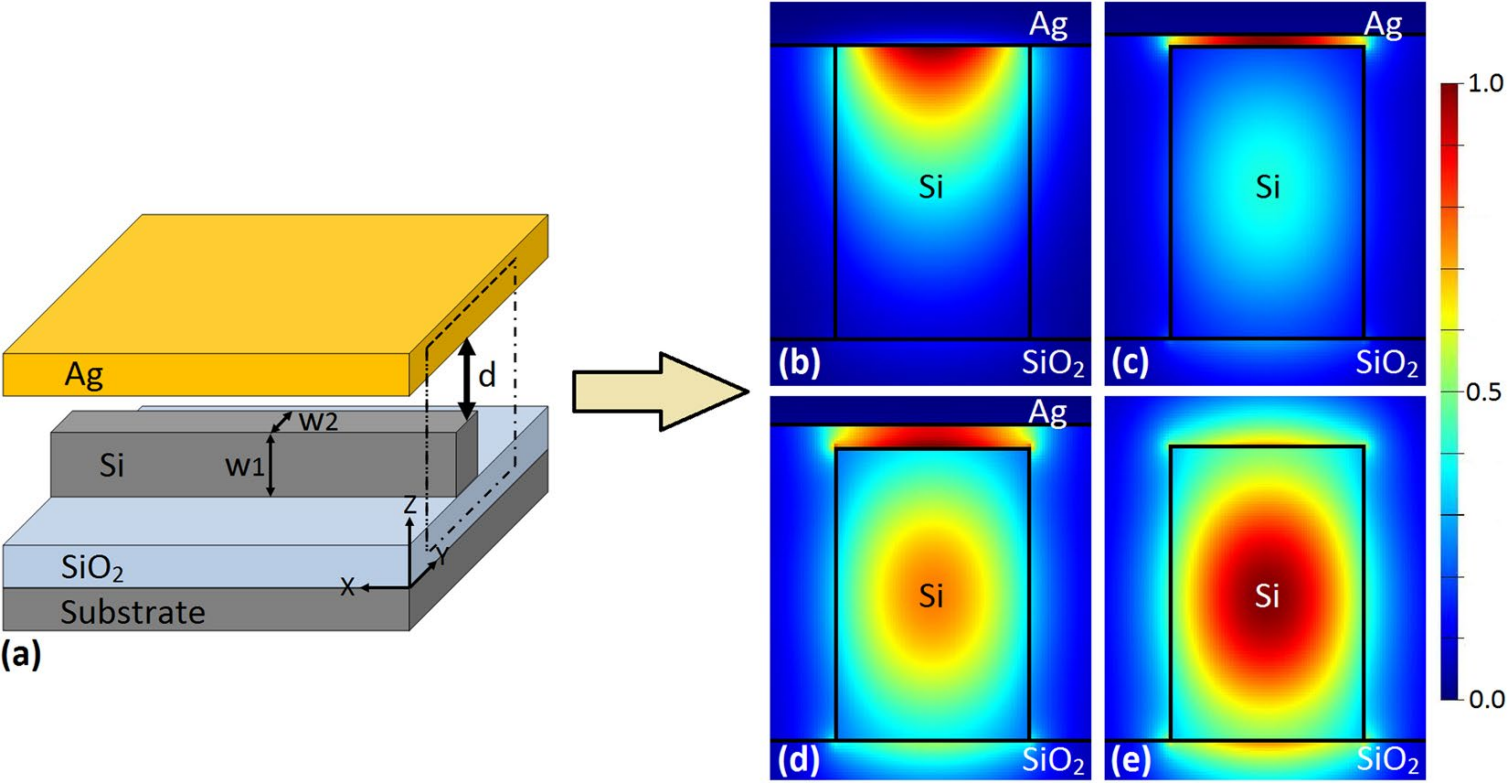
Mode properties in a hybrid plasmonic waveguide. (**a**) Schematic of hybrid plasmonic waveguide (*w*_1_=500 nm, *w*_2_ = 350 nm). (**b**) |*E*_*z*_| distribution of SPP mode with *d* = 0 nm. (**c**,**d**) |*E*_*z*_| distribution of hybrid modes with *d* = 20 and 40 nm, respectively. (**e**) |*E*_*z*_| distribution of dielectric mode with *d* = ∞.

**Figure 2:**
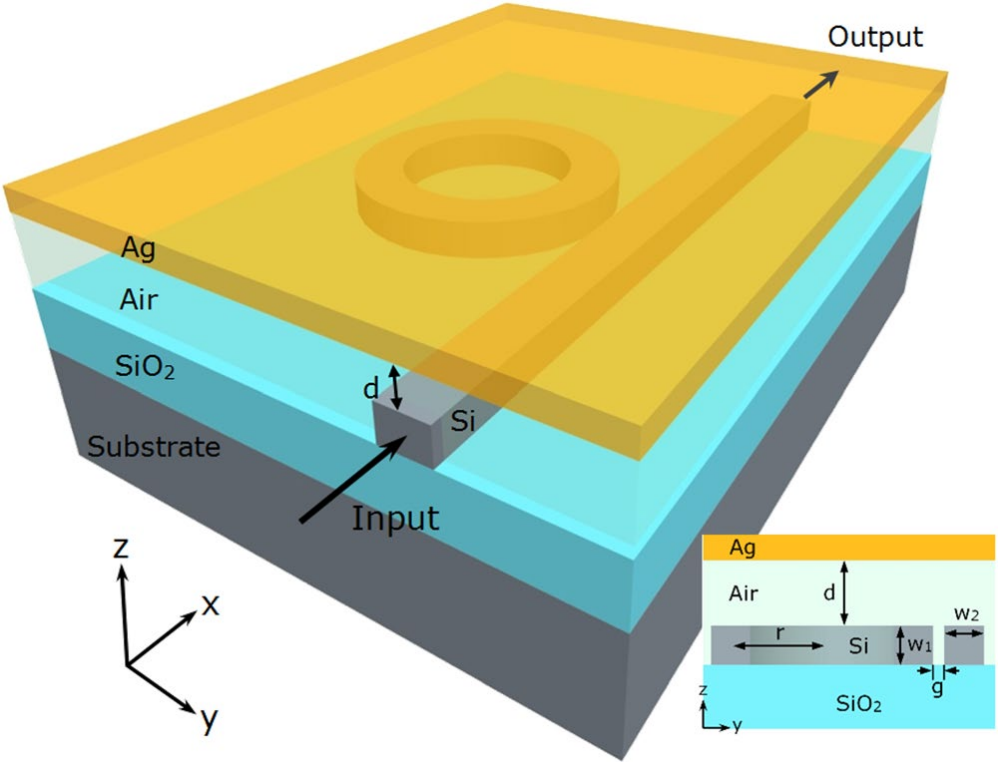
Schematic diagram of hybrid plasmonic microring nano-ruler. (Inset) Definitions of the geometrical parameters. The width and the height of silicon waveguide are *w*_1_ and *w*_2_, respectively. *r* is the radius of microring. The vertical distance d between silver layer and silicon waveguide will be measured by the nano-ruler.

**Figure 3:**
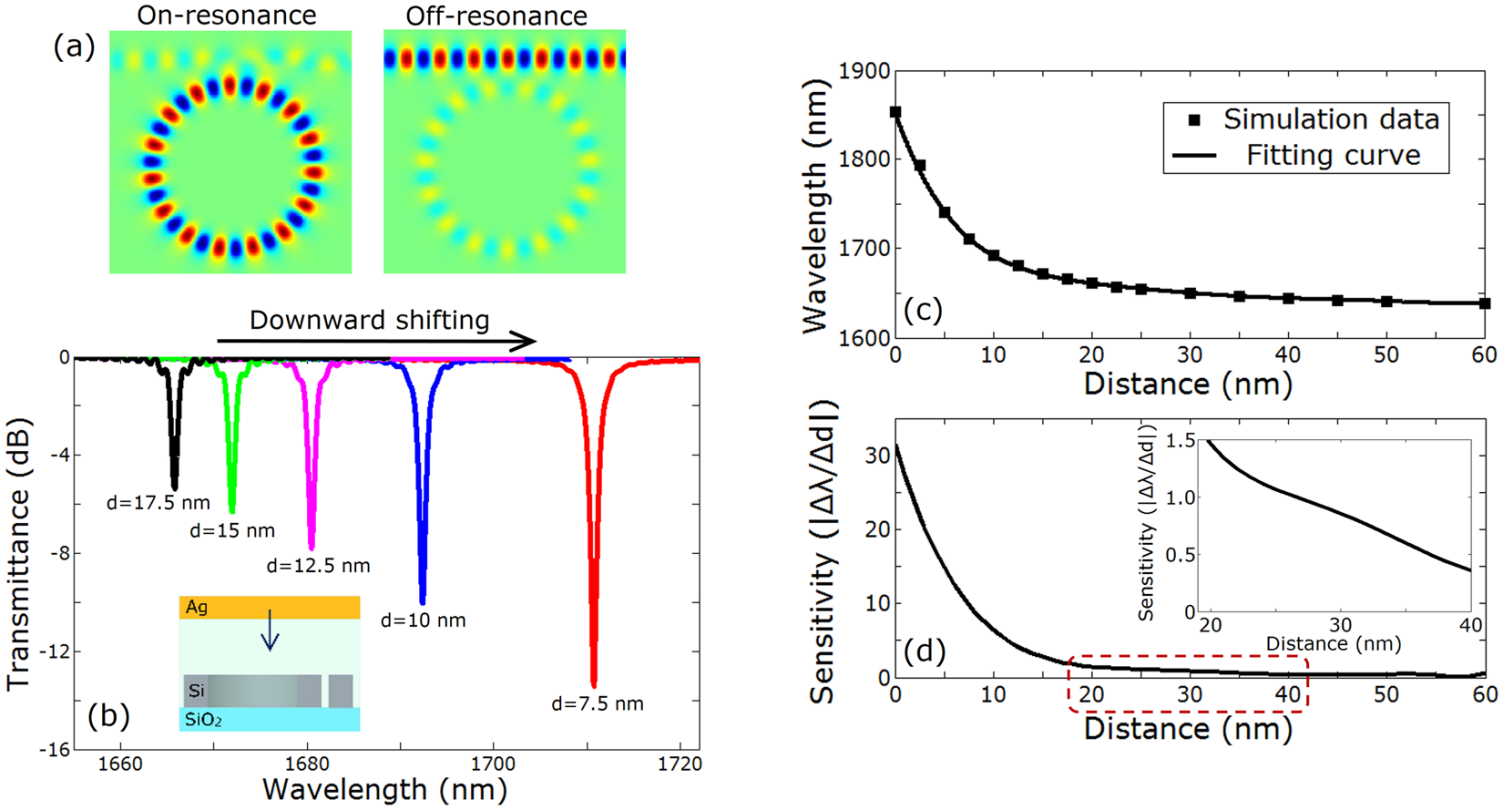
Simulation results of the nano-ruler. (**a**) Distribution of the dominant component *E*_*z*_ in the air gap region under two different operating wavelengths of *λ* = 1661 nm (on-resonance) and *λ* = 1665 nm (off-resonance). The distance *d* = 20 nm. The radius *r* = 1.5 μm. The distance between microring and waveguide is 200 nm. The length of silicon waveguide is 5 μm. (**b**) Transmission spectra under different values of distance varying from 17.5 to 7.5 nm. (**c**) Resonance wavelength vs. distance. (**d**) Sensitivity vs. distance.

To better understand the hybrid mode and its potential use in nano-ruler, we first analyze different mode properties in a hybrid plasmonic waveguide structure, where a silver metal layer (Ag) is suspended in parallel above a silicon waveguide, as shown in Fig. 1(a). The low-index gap region between the metal layer and silicon waveguide is air. The hybrid mode can be regarded as a kind of supermode contributed from SPP and discontinuity of electric field at the interface between silicon and air. To introduce the electric field discontinuity near the top surface between silicon and air, quasi-transverse-magnetic (quasi-TM) mode in silicon waveguide is considered here. Actually, there are three types of modes depending on the distance (*d*) between metal layer and silicon waveguide. Figure 1(b) shows electric field of an SPP mode with *d* = 0. Figure 1(c,d) plot hybrid modes with varied values of *d* (*d* = 20 nm and 40 nm). Figure 1(e) displays a dielectric mode with an infinite value of *d* (i.e. metal layer far away from the silicon waveguide). One can clearly see that the change of the distance effectively affects the mode distribution in the waveguide. Equivalently, the variation of the distance between the metal layer and the silicon waveguide changes the effective mode index, which can cause resonance shift in a microring structure. Consequently, utilizing these mode characteristics, we can design a hybrid plasmonic microring nano-ruler with the three-dimension (3D) structure illustrated in Fig. 2. One can see that the proposed nano-ruler consists of a microring resonator based on silicon-on-insulator (SOI) wafer and a suspending silver layer. The working principle of the proposed nano-ruler relies on the shift of resonance peak by measuring the spectrum at the output side of the silicon waveguide, when the distance between the top surface of microring and the bottom surface of metal layer is changed. Figure 3(a) depicts the distribution of the dominant component *E*_*z*_ in the air gap region under two different operating wavelengths of *λ* = 1661 nm (on-resonance) and *λ* = 1665 nm (off-resonance) calculated by the finite-difference time-domain (FDTD) method with the distance *d* *=* 20 nm. When the distance varies, one can clearly see from Fig. 3(b) that the resonance wavelength shifts. The value of resonance wavelength of the hybrid plasmonic microring increases with the decrease of the distance.

## Performance of the Hybrid Plasmonic Nano-Ruler

We further evaluate the performance of the proposed nano-ruler. Figure 3(c) plots the calculated resonance wavelength as a function of the distance between the top surface of microring and the bottom surface of metal layer. A fitting curve is used to approximate the relationship between the distance to be measured and the resonance wavelength. By solving the first-order derivative of this fitting curve, one can obtain the sensitivity of the nano-ruler which is defined by |*Δλ*/*Δd*|. One can clearly see from Fig. 3(c,d) that the sensitivity increases rapidly as the distance decreases. When the measured distance is larger than 40 nm, a slowly varying sensitivity less than 0.4 is achieved. When the distance is less than 10 nm, the sensitivity is larger than 6.4. Moreover, the sensitivity exceeds 14.8 as the distance is less than 5 nm, which means that a resonance peak shift larger than 14.8 nm can be obtained in the output spectrum as the distance changes 1 nm.

**Figure 4:**
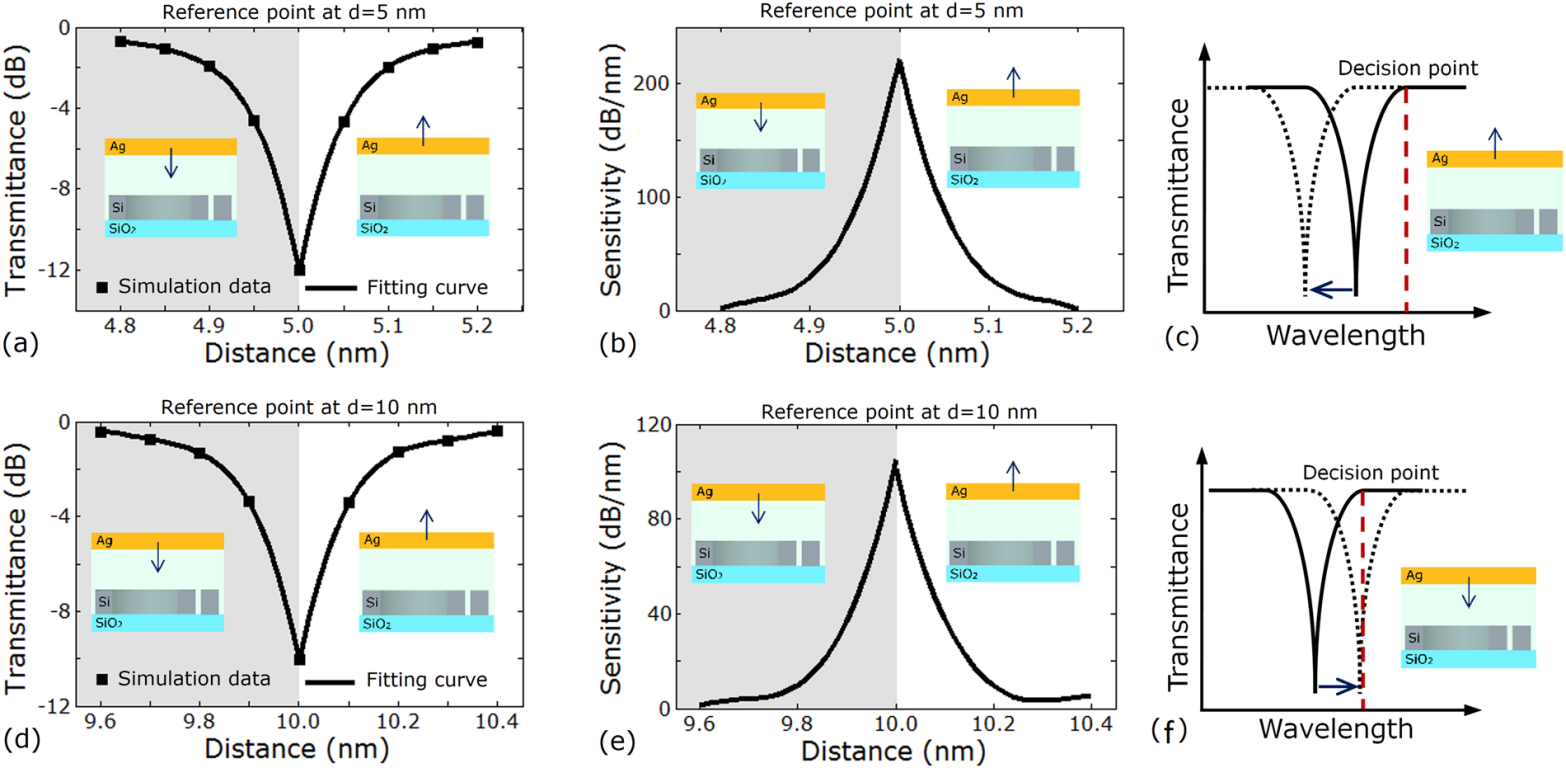
Simulation results of the nano-ruler. (**a**,**d**) Transmittance vs. distance at *d = *5 nm and *d* = 10 nm. (**b**,**e**) Sensitivity vs. distance at *d* = 5 nm and *d* = 10 nm. (**c**,**f**) Schematic diagram of method to judge the direction of distance shifting. (**a**,**b**,**d**,**e**) Grey areas indicate distance downward shifting from reference distance. White areas indicate distance upward shifting from reference distance.

In addition, Fig. 3(b) indicates that transmittance of the output spectrum is able to change with distance by observing the fixed wavelength slice in the spectra, which can also be applied in measuring distance. In this way, it is necessary to find a reference point in the output spectrum. We select the point in the center of one resonance peak in the transmission spectra as the reference point to observe the change of the distance. When the distance is offset from its initial value, the transmittance of the reference point falls on the sides of the resonance peak and therefore changes. Figure 4(a,d) plot the transmittance as a function of the distance between the silver layer and silicon waveguide at reference point of *d* = 5 nm and 10 nm, respectively. No matter the distance shifting downward or upward from its initial value corresponding to the reference point, the transmittance changes rapidly and distributes symmetrically on opposite sides of the reference point. Figure 4(b,e) present the relationship between sensitivity and transmittance at reference point of *d* = 5 nm and 10 nm, respectively. One can see that the sensitivity reaches its maximum near the distance of the reference point. When the distance is 10 nm, the maximum sensitivity gains 105 dB/nm. When the distance is 5 nm, the maximum sensitivity exceeds 200 dB/nm. However, the symmetrical distribution of transmittance as a function of distance makes it difficult to judge the shifting direction of the distance. For example, when the measured transmittance is −4.64 dB at reference distance of 5 nm, it is difficult to distinguish the distance of 4.95 nm and 5.05 nm. Therefore, we design a method to solve this problem shown in Fig. 4(c,f). When recording the transmittance of reference point, we additionally record the transmittance on the right bottom of the peak, called ‘decision point’ (the red line in Fig. 4(c,f)). As distance shifts upward from its initial value, the spectral moves to the left and the transmittance of the decision point remains the same. When distance shifts downward from its initial value, the spectral moves to the right and the transmittance of the decision point changes considerably.

## Measuring Range of the Nano-Ruler

**Figure 5:**
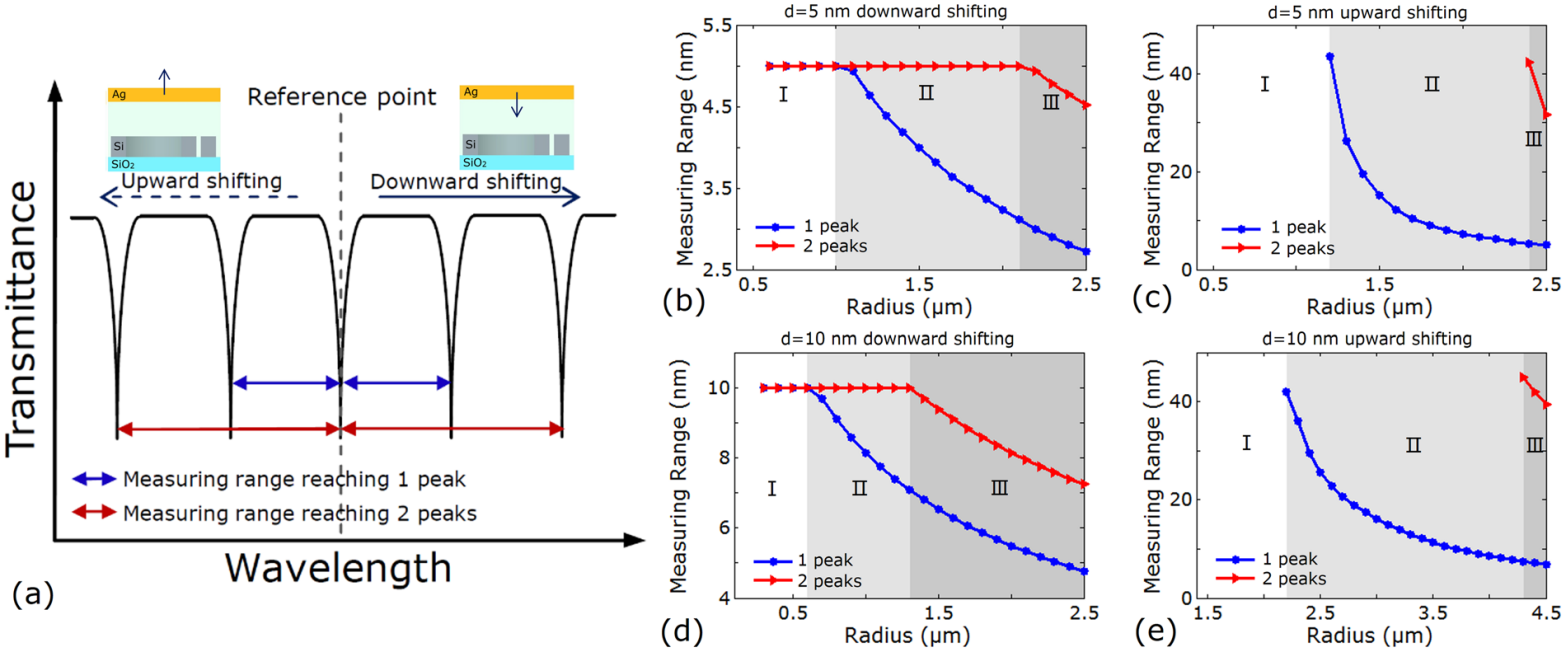
Measuring range of nano-ruler. (**a**) Schematic diagram of measuring range of nano-ruler. (**b**,**d**) Measuring range vs. radius of microring of downward shifting from *d* = 5 nm and *d = *10 nm, respectively. (**c**,**e**) Measuring range vs. radius of microring of upward shifting from *d* = 5 nm and *d* = 10 nm, respectively. (**b**–**e**) Area I, II, and III respectively indicate measuring range reaching 1 peak, 2 peaks and 3 peaks can cover the whole distance shifting range.

Measuring range of sensors based on microring is directly determined by free spectrum range (FSR)^19,20^, which is defined by $\Delta FSR=C/(2\pi {n}_{eff}r)$(where *C* is the speed of light in vacuum, ${n}_{eff}$ is the mode effective index, $r$ is the radius of microring). This is because one can hardly distinguish the adjacent resonance peaks on the transmission spectra if shift of transmission spectra had exceeded FSR. Hence, we define the corresponding distance interval of shifting range between neighbouring resonance peaks on transmission spectra as measuring range under the condition of unsure shifting law of transmission spectra. Figure 5(a) presents the measuring range reaching the nearest resonance peaks (blue line). Reference point is in the center of an arbitrary resonance peak on transmission spectra acting as starting point to measure. Figure 5(b,d) plot the relationship between measuring range and radius of microring as distance downward shifting from the position of *d* = 5 nm and *d* = 10 nm, respectively. When radius is less than 1 μm and 0.6 μm, measuring ranges respectively maintain 5 nm and 10 nm at reference point of *d* = 5 nm and 10 nm, respectively. It means measuring range can cover the whole varitation range of distance as distance downward shifting from the reference position. Figure 5(c,e) depict the relationship between measuring range and radius of microring as distance upward shifting from the position of *d* = 5 nm and *d* = 10 nm, respectively. Measuring range rises with the radius decreasing. When radius is less than 1.2 μm and 2.2 μm, measuring ranges turn into infinite at reference distance *d* = 5 nm and 10 nm, respectively. It also means measuring range can cover the whole varitation range of distance as distance upward shifting from the reference position. Furthermore, if varying rule of transmission spectra and distance had been grasped, measuring range reaching further resonance peaks is available. Figure 5 also displays the measuring range reaching 2 peaks (red lines) on the spectra and pratical distance. The critical values of radius where measuring range can cover the whole distance can rise to 2.1 μm, 2.4 μm, 1.3 μm 4.3 μm in Fig. 5(b–e), respectively, which means that the measuring range improves considerably.

## Discussion

These simulation results indicate that hybrid plasmonic nano-ruler has various ways to measure the distance between metal layer and silicon waveguide. Whether it is watching the shift of transmission spectra or recording the change of output transmittance, sensitivities of each approach are all altering with the varying of distance all the times. In the first approach, sensitivity increases with the decrease of the distance. In the second approach, sensitivity is relatively high as distance is near the distance of reference point while it begins to reduce as distance is leaving away from the distance of reference point. Although the sensitivities of the nano-ruler do not have fixed value, this phenomenon indicates that sensitivity is self-adaption to difference distance, which is important for nano-ruler. Moreover, if we associate the simulation results with existing equipment, we can estimate the resolution of the nano-ruler. Assuming the resolutions of optical spectrum analyzers and optical power meter are 20 pm (e.g. Yokogawa optical spectrum analyzer AQ6370D) and 0.01 dBm (e.g. Yokogawa Optical Power Meter AQ2170), respectively, the evaluated resolution of nano-ruler can reach 1.35 pm at *d* = 5 nm in the first approach and about 0.05 pm near the reference distance 5 nm in the second approach. There exists an order of magnitude of the gap between two resolutions calculated by two approaches, which indicates that they can be applied in different occasions. For instance, the approach of lower resolution can be used to preliminarily measure distance to get a value of low accuracy. Then we can further measure distance accurately using the approach with higher resolution. This combination measurement scheme tremendously enhances the working performance of nano-ruler. In addition, although the proposed nano-ruler is only analyzed in simulation and calculation, it can be made out in reality by bonding the metal layer on the target object or fabricating the pillars to support the metal layer above the microring. For example, by bonding the metal layer on the object 1 and the microring on the object 2, the minor changes of the distance between the two objects can be measured. In another scene, the metal layer is supported by the pillars. When a small force is added on the metal layer, it can be measured by the deformation of the metal layer.

In summary, a novel hybrid plasmonic microring nano-ruler that employs the sensitive hybrid mode property and the microring resonator has been designed and simulated. Two working mechanisms of the proposed nano-ruler are discussed and the simulated results present attractive working performance such as relatively high sensitivity and wide measuring range. This concept of nano-ruler has broad application prospects: 1) using vertical distance sensitiveness, it can detect surface morphology of analytes^21–23^; 2) in the field of biochemical, it can monitor the activities of chemical reactions and biological processed by measuring the size of analytes between metal and silicon waveguide or detect macromolecular transformations in three-dimension by joining three nano-ruler in three orthogonal directions^7,8^; 3) by linking the metal layer to external force, it can be even applied in small force detection^24^.

## Methods

### Simulation

We use a three-dimension finite difference time domain (FDTD) method to simulate the spread of electromagnetic field in our nano-ruler structure. Local grid refinement technology is used in the region between silicon waveguide and metal layer where the grid size is 1 nm as the distance less than 10 nm to ensure the accuracy of simulation and decrease the calculation time. The light source is placed at the input port and two monitors perpendicular to the spread direction of light field are respectively placed at input port and output port to record the distribution of electromagnetic field and power. By comparing the input and output power, we numerically calculate the transmission spectra. Another monitor parallel to the spread direction of light field in the air region to record the resonance mode distribution. The whole area is surrounded by a perfectly matched layer (PML). The distance between bus waveguide and microring is set to 200 nm in the simulation to obtain the best coupling efficiency.

### Curve Fitting

We use a six-order curve to fit the function between resonance wavelength and distance given by:

1 $$\lambda (d)=\sum _{i=0}^{6}{a}_{i}{d}^{i}$$

where $\lambda (d)$ is resonance wavelength function, $d$ is the distance between metal layer and silicon waveguide, ${a}_{i}$ is the coefficient of *i*-order variable. By calculating one-order derivative of this fitting curve we get the sensitivity function:

2 $$s(d)=|\frac{\partial \lambda }{\partial d}|=|i\sum _{i=0}^{6}{a}_{i}{d}^{i-1}|$$

### Coupled mode theory

According to the coupled mode theory^11^, the supermode can be regarded as the linear superposition of basis vectors, which are uncoupled dielectric mode and SPP mode in this paper. Dielectric mode is the eigenmode in the silicon waveguide. SPP mode is the eigenmode near the surface between the metal and air. Therefore, the mode in the hybrid SPP waveguide can be expressed as^11,25^:

3 $$\Psi =a{\Psi }_{d}+b{\Psi }_{SPP}$$

where $a$ and $b={(1-|a{|}^{2})}^{1/2}$ are the mode amplitudes of the dielectric mode and the SPP mode respectively. Then the relationship between dielectric mode, SPP mode and hybrid mode can be written as:

4 $$a{n}_{d}+bV=a{n}_{hyb}$$

5 $$aV+b{n}_{SPP}=b{n}_{hyb}$$

where ${n}_{d}$, ${n}_{spp}$, and ${n}_{hyb}$ are effective mode indices of dielectric mode, SPP mode and hybrid mode, respectively. *V* is the coupling strength between dielectric and SPP modes, which is corresponding to the distance between metal layer and dielectric waveguide. The varying of distance between metal layer and dielectric waveguide can affect the effective index of hybrid SPP mode. When the dielectric waveguide is approaching the metal layer, the coupling strength *V* is increasing that part of dielectric mode is coupled into SPP mode near the surface between metal and air. In this situation, the electric field in the air gap between metal layer and silicon waveguide is a superposition of electric fields of dielectric mode and SPP mode.
